# 1,4-Ditosyl-1,4-diazepane

**DOI:** 10.1107/S1600536812020958

**Published:** 2012-05-12

**Authors:** Shuang-Hua Yang, Zhi-Wei Zhai

**Affiliations:** a Department of Environment Engineering and Chemistry, Luoyang Institute of Science and Technology, Luoyang 471023, People’s Republic of China

## Abstract

In the title compound, C_19_H_24_N_2_O_4_S_2_, the dihedral angle formed by the benzene rings is 82.88 (7)°, and the mol­ecular conformation is enforced by weak intra­molecular C—H⋯O contacts. Two C atoms of the 1,4-diazepane ring are disordered over two sets of sites with a refined occupancy ratio of 0.534 (13):0.466 (13). In the crystal, mol­ecules are linked by weak inter­molecular C—H⋯O inter­actions into chains parallel to the *a* axis.

## Related literature

For related structures, see: Romba *et al.* (2002 ▶). For the biological activity of heterocyclic compounds, see: Xu *et al.* (2006 ▶); Yu *et al.* (2009 ▶).
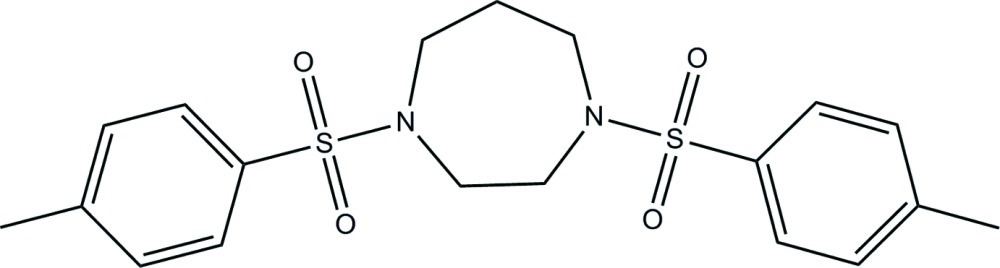


## Experimental

### 

#### Crystal data

C_19_H_24_N_2_O_4_S_2_
*M* *_r_* = 408.52
Orthorhombic, 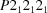

*a* = 6.3407 (13) Å
*b* = 10.367 (2) Å
*c* = 30.516 (6) Å
*V* = 2005.9 (7) Å^3^
*Z* = 4
Mo *K*α radiation
μ = 0.29 mm^−1^
*T* = 173 K
0.20 × 0.20 × 0.10 mm

#### Data collection

Rigaku Mercury CCD/AFC diffractometer
Absorption correction: multi-scan (*CrystalClear*; Rigaku, 2007 ▶) *T*_min_ = 0.944, *T*_max_ = 0.971
11747 measured reflections
3531 independent reflections
3430 reflections with *I* > 2σ(*I*)
*R*_int_ = 0.035

#### Refinement

*R*[*F* ^2^ > 2σ(*F* ^2^)] = 0.036
*wR*(*F* ^2^) = 0.083
*S* = 1.07
3531 reflections
265 parameters
6 restraints
H-atom parameters constrained
Δρ_max_ = 0.13 e Å^−3^
Δρ_min_ = −0.20 e Å^−3^
Absolute structure: Flack (1983 ▶), 1442 Friedel pairs
Flack parameter: −0.03 (7)

### 

Data collection: *CrystalClear* (Rigaku, 2007 ▶); cell refinement: *CrystalClear*; data reduction: *CrystalClear*; program(s) used to solve structure: *SHELXS97* (Sheldrick, 2008 ▶); program(s) used to refine structure: *SHELXL97* (Sheldrick, 2008 ▶); molecular graphics: *SHELXTL* (Sheldrick, 2008 ▶); software used to prepare material for publication: *SHELXTL*.

## Supplementary Material

Crystal structure: contains datablock(s) I, global. DOI: 10.1107/S1600536812020958/rz2746sup1.cif

Structure factors: contains datablock(s) I. DOI: 10.1107/S1600536812020958/rz2746Isup2.hkl

Supplementary material file. DOI: 10.1107/S1600536812020958/rz2746Isup3.cml

Additional supplementary materials:  crystallographic information; 3D view; checkCIF report

## Figures and Tables

**Table 1:** Hydrogen-bond geometry (Å, °)

*D*—H⋯*A*	*D*—H	H⋯*A*	*D*⋯*A*	*D*—H⋯*A*
C10—H10*B*⋯O3	0.97	2.40	2.886 (3)	110
C12—H12*A*⋯O1	0.97	2.39	2.878 (3)	111
C10—H10*B*⋯O4^i^	0.97	2.52	3.142 (3)	122
C12—H12*A*⋯O2^i^	0.97	2.50	3.035 (3)	115

Symmetry code: (i) 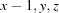.

**Fig. 1.**
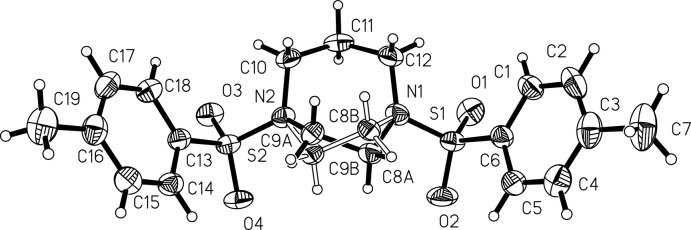
The molecular structure of the title compound, with 40% probability displacement ellipsoids.

## supplementary crystallographic information

### Comment

Many heterocyclic compounds have been widely used as potent and broad-spectrum fungicides (Xu *et al.*, 2006; Yu *et al.*, 2009). In order to search for new heterocylic compounds with higher biological activities, we synthesized the title compound and describe its structure herein.

In title compound (Fig. 1), all bond lengths are normal and in a good agreement with those reported for related compounds (Romba *et al.*, 2002). Two atoms (C8 and C9) of the 1,4-diazepane ring are disordered over two orientations with refined occupancy ratio of 0.466 (13)/0.534 (13). The dihedral angle formed by the phenyl rings is 82.88 (7)°. The molecular conformation is stabilized by intramolecular C—H···O hydrogen bonds (Table 1). In the crystal packing, molecules are linked by intermolecular C—H···O hydrogen bonds to form chains parallel to the *a* axis.

### Experimental

NaH (1.9 g, 0.08 mol) was dissolved in 30 ml DMF and cooled with an ice bath, then *N*,*N*'-di(*p*-toluenesulfonyl)ethane-1,2-diamine (7.4 g, 0.02 mol) in 10 ml DMF was added dropwise to the solution. After stirring 30 min, 1,3-dibromopropane in 5 ml DMF was added dropwise and the resulting suspension stirred overnight at room temperature. The residue was quenched by saturated NH_4_Cl and extracted with ethyl acetate (3 × 200 ml). The combined organic layer was washed with saturated NaCl and dried over sodium sulfate. The solvent was removed and the residue was recrystallized from EtOH/DMF (5:1 *v*/*v*) to give the title compound (6.7 g, 82%). Single crystals suitable for X-ray measurements were obtained by recrystallization from acetonitrile at room temperature.

### Refinement

H atoms were positioned geometrically and refined using a riding model, with C—H = 0.95 or 0.99 Å and with *U*_iso_(H) = 1.2 times *U*_eq_(C) for methylene H atoms and 1.5*U*_eq_(C) for the methyl H atoms. Distance constraints (N—C = 1.47 (1) Å; C—C = 1.52 (1) Å) were applied to the disordered atoms C8 and C9.

### Crystal data

**Table:**

C_19_H_24_N_2_O_4_S_2_	*F*(000) = 864
*M**_r_* = 408.52	*D*_x_ = 1.353 Mg m^−^^3^
Orthorhombic, *P*2_1_2_1_2_1_	Mo *K*α radiation, λ = 0.71073 Å
Hall symbol: P 2ac 2ab	Cell parameters from 7269 reflections
*a* = 6.3407 (13) Å	θ = 1.7–27.5°
*b* = 10.367 (2) Å	µ = 0.29 mm^−^^1^
*c* = 30.516 (6) Å	*T* = 173 K
*V* = 2005.9 (7) Å^3^	Block, colourless
*Z* = 4	0.20 × 0.20 × 0.10 mm

### Data collection

**Table:**

Rigaku Mercury CCD/AFC diffractometer	3531 independent reflections
Radiation source: Sealed Tube	3430 reflections with *I* > 2σ(*I*)
Graphite Monochromator monochromator	*R*_int_ = 0.035
φ and ω scans	θ_max_ = 25.0°, θ_min_ = 2.8°
Absorption correction: multi-scan (*CrystalClear*; Rigaku, 2007)	*h* = −7→7
*T*_min_ = 0.944, *T*_max_ = 0.971	*k* = −8→12
11747 measured reflections	*l* = −36→35

### Refinement

**Table:**

Refinement on *F*^2^	Secondary atom site location: difference Fourier map
Least-squares matrix: full	Hydrogen site location: inferred from neighbouring sites
*R*[*F*^2^ > 2σ(*F*^2^)] = 0.036	H-atom parameters constrained
*wR*(*F*^2^) = 0.083	*w* = 1/[σ^2^(*F*_o_^2^) + (0.035*P*)^2^ + 0.4874*P*] where *P* = (*F*_o_^2^ + 2*F*_c_^2^)/3
*S* = 1.07	(Δ/σ)_max_ < 0.001
3531 reflections	Δρ_max_ = 0.13 e Å^−^^3^
265 parameters	Δρ_min_ = −0.20 e Å^−^^3^
6 restraints	Absolute structure: Flack (1983), 1442 Friedel pairs
Primary atom site location: structure-invariant direct methods	Flack parameter: −0.03 (7)

### Special details

**Table:**

Geometry. All e.s.d.'s (except the e.s.d. in the dihedral angle between two l.s. planes) are estimated using the full covariance matrix. The cell e.s.d.'s are taken into account individually in the estimation of e.s.d.'s in distances, angles and torsion angles; correlations between e.s.d.'s in cell parameters are only used when they are defined by crystal symmetry. An approximate (isotropic) treatment of cell e.s.d.'s is used for estimating e.s.d.'s involving l.s. planes.
Refinement. Refinement of *F*^2^ against ALL reflections. The weighted *R*-factor *wR* and goodness of fit *S* are based on *F*^2^, conventional *R*-factors *R* are based on *F*, with *F* set to zero for negative *F*^2^. The threshold expression of *F*^2^ > σ(*F*^2^) is used only for calculating *R*-factors(gt) *etc*. and is not relevant to the choice of reflections for refinement. *R*-factors based on *F*^2^ are statistically about twice as large as those based on *F*, and *R*- factors based on ALL data will be even larger.

### Fractional atomic coordinates and isotropic or equivalent isotropic displacement parameters (Å^2^)

**Table:**

	*x*	*y*	*z*	*U*_iso_*/*U*_eq_	Occ. (<1)
S1	0.84854 (9)	−0.00702 (6)	0.40081 (2)	0.04437 (16)	
S2	0.83732 (9)	0.12928 (5)	0.22154 (2)	0.03921 (15)	
O1	0.7421 (3)	0.08375 (18)	0.42783 (6)	0.0594 (5)	
O2	1.0685 (3)	0.0089 (2)	0.39251 (7)	0.0716 (6)	
O3	0.7251 (3)	0.24688 (15)	0.21378 (6)	0.0501 (4)	
O4	1.0600 (2)	0.13432 (18)	0.22902 (6)	0.0532 (5)	
N1	0.7323 (3)	−0.0080 (2)	0.35377 (6)	0.0439 (5)	
N2	0.7313 (3)	0.06018 (17)	0.26359 (6)	0.0388 (5)	
C1	0.6335 (4)	−0.1951 (3)	0.44536 (8)	0.0511 (6)	
H1	0.5214	−0.1374	0.4464	0.061*	
C2	0.6164 (5)	−0.3155 (3)	0.46458 (8)	0.0603 (8)	
H2	0.4911	−0.3386	0.4783	0.072*	
C3	0.7831 (6)	−0.4029 (3)	0.46375 (8)	0.0618 (8)	
C4	0.9649 (5)	−0.3682 (3)	0.44218 (9)	0.0589 (7)	
H4	1.0765	−0.4263	0.4408	0.071*	
C5	0.9853 (4)	−0.2489 (3)	0.42246 (8)	0.0495 (6)	
H5	1.1091	−0.2274	0.4079	0.059*	
C6	0.8205 (4)	−0.1615 (2)	0.42453 (7)	0.0419 (5)	
C8A	0.8715 (15)	−0.0801 (9)	0.3232 (2)	0.044 (2)	0.466 (13)
H8A1	0.8801	−0.1695	0.3323	0.053*	0.466 (13)
H8A2	1.0125	−0.0438	0.3241	0.053*	0.466 (13)
C9A	0.7867 (18)	−0.0729 (7)	0.2768 (3)	0.040 (2)	0.466 (13)
H9A1	0.8920	−0.1065	0.2568	0.048*	0.466 (13)
H9A2	0.6624	−0.1270	0.2745	0.048*	0.466 (13)
C8B	0.7774 (14)	−0.1092 (6)	0.31983 (19)	0.0377 (15)	0.534 (13)
H8B1	0.6480	−0.1544	0.3126	0.045*	0.534 (13)
H8B2	0.8765	−0.1714	0.3316	0.045*	0.534 (13)
C9B	0.8687 (11)	−0.0489 (7)	0.2785 (3)	0.0353 (17)	0.534 (13)
H9B1	1.0098	−0.0173	0.2844	0.042*	0.534 (13)
H9B2	0.8778	−0.1135	0.2556	0.042*	0.534 (13)
C10	0.5022 (4)	0.0686 (3)	0.26975 (8)	0.0517 (7)	
H10A	0.4390	−0.0144	0.2633	0.062*	
H10B	0.4447	0.1312	0.2494	0.062*	
C11	0.4459 (4)	0.1078 (3)	0.31613 (9)	0.0518 (7)	
H11A	0.5150	0.1891	0.3226	0.062*	
H11B	0.2950	0.1226	0.3175	0.062*	
C12	0.5040 (4)	0.0126 (3)	0.35127 (8)	0.0536 (7)	
H12A	0.4535	0.0438	0.3793	0.064*	
H12B	0.4350	−0.0691	0.3453	0.064*	
C13	0.7984 (4)	0.0278 (2)	0.17594 (7)	0.0394 (5)	
C14	0.9546 (4)	−0.0588 (2)	0.16374 (8)	0.0449 (6)	
H14	1.0803	−0.0630	0.1794	0.054*	
C15	0.9215 (4)	−0.1392 (3)	0.12792 (8)	0.0515 (6)	
H15	1.0266	−0.1968	0.1196	0.062*	
C16	0.7342 (5)	−0.1350 (3)	0.10436 (9)	0.0521 (6)	
C17	0.5795 (4)	−0.0506 (3)	0.11791 (9)	0.0543 (7)	
H17	0.4521	−0.0486	0.1028	0.065*	
C18	0.6080 (4)	0.0313 (2)	0.15328 (8)	0.0469 (6)	
H18	0.5015	0.0877	0.1618	0.056*	
C19	0.7052 (6)	−0.2180 (3)	0.06426 (9)	0.0691 (9)	
H19A	0.5637	−0.2509	0.0636	0.104*	
H19B	0.8030	−0.2887	0.0652	0.104*	
H19C	0.7305	−0.1673	0.0385	0.104*	
C7	0.7658 (6)	−0.5318 (3)	0.48659 (10)	0.0877 (12)	
H7A	0.7705	−0.5997	0.4652	0.131*	
H7B	0.6349	−0.5359	0.5023	0.131*	
H7C	0.8810	−0.5415	0.5067	0.131*	

### Atomic displacement parameters (Å^2^)

**Table:**

	*U*^11^	*U*^22^	*U*^33^	*U*^12^	*U*^13^	*U*^23^
S1	0.0326 (3)	0.0467 (3)	0.0537 (3)	−0.0031 (3)	0.0021 (3)	0.0001 (3)
S2	0.0305 (3)	0.0333 (3)	0.0538 (3)	−0.0003 (2)	−0.0042 (3)	0.0032 (3)
O1	0.0676 (13)	0.0522 (11)	0.0584 (11)	0.0016 (9)	−0.0065 (9)	−0.0171 (9)
O2	0.0299 (9)	0.0798 (14)	0.1052 (15)	−0.0070 (10)	0.0024 (10)	0.0327 (13)
O3	0.0498 (10)	0.0296 (8)	0.0710 (12)	0.0033 (7)	−0.0041 (9)	0.0093 (8)
O4	0.0271 (8)	0.0608 (11)	0.0717 (12)	−0.0036 (8)	−0.0061 (8)	−0.0080 (10)
N1	0.0411 (11)	0.0450 (11)	0.0457 (10)	0.0089 (9)	0.0087 (9)	−0.0023 (10)
N2	0.0373 (11)	0.0326 (10)	0.0464 (11)	0.0048 (8)	−0.0031 (9)	0.0025 (9)
C1	0.0479 (15)	0.0597 (16)	0.0458 (13)	−0.0042 (13)	0.0092 (12)	−0.0037 (12)
C2	0.073 (2)	0.0680 (18)	0.0401 (14)	−0.0238 (16)	0.0104 (14)	−0.0042 (13)
C3	0.094 (2)	0.0522 (16)	0.0395 (14)	−0.0121 (16)	−0.0093 (15)	−0.0031 (12)
C4	0.077 (2)	0.0505 (15)	0.0497 (15)	0.0081 (16)	−0.0049 (14)	−0.0054 (13)
C5	0.0482 (15)	0.0590 (16)	0.0413 (14)	0.0046 (13)	0.0025 (12)	−0.0031 (12)
C6	0.0394 (13)	0.0492 (14)	0.0370 (12)	−0.0026 (11)	0.0018 (11)	−0.0036 (10)
C8A	0.035 (4)	0.050 (4)	0.048 (4)	0.011 (4)	0.005 (3)	0.006 (3)
C9A	0.046 (5)	0.031 (3)	0.043 (4)	−0.001 (3)	−0.004 (4)	0.001 (3)
C8B	0.036 (4)	0.041 (3)	0.036 (3)	0.002 (3)	0.003 (3)	−0.005 (2)
C9B	0.035 (4)	0.030 (3)	0.041 (3)	0.006 (2)	−0.001 (3)	0.001 (3)
C10	0.0334 (13)	0.0642 (17)	0.0576 (16)	−0.0106 (12)	−0.0099 (11)	0.0107 (14)
C11	0.0268 (11)	0.0530 (16)	0.0758 (18)	0.0059 (11)	−0.0004 (12)	−0.0106 (13)
C12	0.0353 (13)	0.0765 (19)	0.0491 (14)	−0.0133 (13)	0.0033 (11)	−0.0035 (15)
C13	0.0356 (12)	0.0383 (12)	0.0442 (12)	−0.0016 (10)	−0.0024 (10)	0.0101 (10)
C14	0.0402 (13)	0.0438 (13)	0.0506 (14)	0.0029 (11)	−0.0007 (12)	0.0061 (12)
C15	0.0561 (16)	0.0442 (14)	0.0543 (15)	0.0045 (13)	0.0055 (13)	0.0047 (13)
C16	0.0640 (17)	0.0446 (13)	0.0477 (14)	−0.0054 (13)	−0.0038 (13)	0.0051 (13)
C17	0.0542 (16)	0.0577 (16)	0.0511 (14)	−0.0062 (13)	−0.0158 (13)	0.0090 (13)
C18	0.0413 (14)	0.0464 (14)	0.0529 (14)	0.0032 (11)	−0.0066 (11)	0.0019 (11)
C19	0.092 (2)	0.0543 (17)	0.0613 (18)	−0.0067 (17)	−0.0096 (17)	−0.0056 (14)
C7	0.140 (4)	0.063 (2)	0.0591 (18)	−0.028 (2)	−0.021 (2)	0.0131 (15)

### Geometric parameters (Å, º)

**Table:**

S1—O1	1.4216 (19)	C8B—C9B	1.522 (7)
S1—O2	1.4273 (18)	C8B—H8B1	0.9700
S1—N1	1.614 (2)	C8B—H8B2	0.9700
S1—C6	1.766 (2)	C9B—H9B1	0.9700
S2—O4	1.4313 (17)	C9B—H9B2	0.9700
S2—O3	1.4313 (16)	C10—C11	1.515 (4)
S2—N2	1.616 (2)	C10—H10A	0.9700
S2—C13	1.762 (2)	C10—H10B	0.9700
N1—C12	1.465 (3)	C11—C12	1.504 (4)
N1—C8A	1.486 (6)	C11—H11A	0.9700
N1—C8B	1.502 (5)	C11—H11B	0.9700
N2—C10	1.467 (3)	C12—H12A	0.9700
N2—C9A	1.480 (7)	C12—H12B	0.9700
N2—C9B	1.499 (6)	C13—C14	1.388 (3)
C1—C2	1.384 (4)	C13—C18	1.392 (3)
C1—C6	1.389 (4)	C14—C15	1.391 (4)
C1—H1	0.9300	C14—H14	0.9300
C2—C3	1.392 (4)	C15—C16	1.389 (4)
C2—H2	0.9300	C15—H15	0.9300
C3—C4	1.375 (4)	C16—C17	1.378 (4)
C3—C7	1.511 (4)	C16—C19	1.507 (4)
C4—C5	1.381 (4)	C17—C18	1.385 (4)
C4—H4	0.9300	C17—H17	0.9300
C5—C6	1.385 (3)	C18—H18	0.9300
C5—H5	0.9300	C19—H19A	0.9600
C8A—C9A	1.516 (8)	C19—H19B	0.9600
C8A—H8A1	0.9700	C19—H19C	0.9600
C8A—H8A2	0.9700	C7—H7A	0.9600
C9A—H9A1	0.9700	C7—H7B	0.9600
C9A—H9A2	0.9700	C7—H7C	0.9600
			
O1—S1—O2	119.36 (13)	C9B—C8B—H8B2	109.5
O1—S1—N1	107.66 (11)	H8B1—C8B—H8B2	108.0
O2—S1—N1	106.80 (12)	N2—C9B—C8B	109.9 (5)
O1—S1—C6	108.33 (12)	N2—C9B—H9B1	109.7
O2—S1—C6	106.02 (12)	C8B—C9B—H9B1	109.7
N1—S1—C6	108.25 (11)	N2—C9B—H9B2	109.7
O4—S2—O3	119.05 (11)	C8B—C9B—H9B2	109.7
O4—S2—N2	107.45 (11)	H9B1—C9B—H9B2	108.2
O3—S2—N2	107.59 (10)	N2—C10—C11	111.7 (2)
O4—S2—C13	106.62 (11)	N2—C10—H10A	109.3
O3—S2—C13	107.95 (11)	C11—C10—H10A	109.3
N2—S2—C13	107.71 (10)	N2—C10—H10B	109.3
C12—N1—C8A	128.8 (4)	C11—C10—H10B	109.3
C12—N1—C8B	104.7 (4)	H10A—C10—H10B	107.9
C12—N1—S1	119.77 (16)	C12—C11—C10	115.6 (2)
C8A—N1—S1	106.9 (3)	C12—C11—H11A	108.4
C8B—N1—S1	122.1 (3)	C10—C11—H11A	108.4
C10—N2—C9A	104.8 (5)	C12—C11—H11B	108.4
C10—N2—C9B	125.6 (3)	C10—C11—H11B	108.4
C10—N2—S2	119.14 (16)	H11A—C11—H11B	107.4
C9A—N2—S2	122.1 (5)	N1—C12—C11	112.0 (2)
C9B—N2—S2	109.5 (3)	N1—C12—H12A	109.2
C2—C1—C6	119.2 (3)	C11—C12—H12A	109.2
C2—C1—H1	120.4	N1—C12—H12B	109.2
C6—C1—H1	120.4	C11—C12—H12B	109.2
C1—C2—C3	121.3 (3)	H12A—C12—H12B	107.9
C1—C2—H2	119.3	C14—C13—C18	120.2 (2)
C3—C2—H2	119.3	C14—C13—S2	119.87 (18)
C4—C3—C2	118.4 (3)	C18—C13—S2	119.90 (19)
C4—C3—C7	120.9 (3)	C13—C14—C15	119.4 (2)
C2—C3—C7	120.8 (3)	C13—C14—H14	120.3
C3—C4—C5	121.4 (3)	C15—C14—H14	120.3
C3—C4—H4	119.3	C16—C15—C14	121.1 (3)
C5—C4—H4	119.3	C16—C15—H15	119.4
C4—C5—C6	119.7 (3)	C14—C15—H15	119.4
C4—C5—H5	120.1	C17—C16—C15	118.3 (3)
C6—C5—H5	120.1	C17—C16—C19	121.3 (3)
C5—C6—C1	120.0 (2)	C15—C16—C19	120.5 (3)
C5—C6—S1	119.9 (2)	C16—C17—C18	122.0 (3)
C1—C6—S1	120.1 (2)	C16—C17—H17	119.0
N1—C8A—C9A	110.6 (7)	C18—C17—H17	119.0
N1—C8A—H8A1	109.5	C17—C18—C13	119.0 (2)
C9A—C8A—H8A1	109.5	C17—C18—H18	120.5
N1—C8A—H8A2	109.5	C13—C18—H18	120.5
C9A—C8A—H8A2	109.5	C16—C19—H19A	109.5
H8A1—C8A—H8A2	108.1	C16—C19—H19B	109.5
N2—C9A—C8A	112.6 (6)	H19A—C19—H19B	109.5
N2—C9A—H9A1	109.1	C16—C19—H19C	109.5
C8A—C9A—H9A1	109.1	H19A—C19—H19C	109.5
N2—C9A—H9A2	109.1	H19B—C19—H19C	109.5
C8A—C9A—H9A2	109.1	C3—C7—H7A	109.5
H9A1—C9A—H9A2	107.8	C3—C7—H7B	109.5
N1—C8B—C9B	110.9 (6)	H7A—C7—H7B	109.5
N1—C8B—H8B1	109.5	C3—C7—H7C	109.5
C9B—C8B—H8B1	109.5	H7A—C7—H7C	109.5
N1—C8B—H8B2	109.5	H7B—C7—H7C	109.5
			
O1—S1—N1—C12	−34.0 (3)	S1—N1—C8A—C9A	−173.6 (8)
O2—S1—N1—C12	−163.3 (2)	C10—N2—C9A—C8A	−98.8 (10)
C6—S1—N1—C12	82.9 (2)	C9B—N2—C9A—C8A	60.8 (13)
O1—S1—N1—C8A	167.7 (5)	S2—N2—C9A—C8A	121.8 (9)
O2—S1—N1—C8A	38.4 (5)	N1—C8A—C9A—N2	48.4 (15)
C6—S1—N1—C8A	−75.4 (5)	C12—N1—C8B—C9B	101.7 (7)
O1—S1—N1—C8B	−168.7 (4)	C8A—N1—C8B—C9B	−57.8 (9)
O2—S1—N1—C8B	62.0 (4)	S1—N1—C8B—C9B	−118.0 (6)
C6—S1—N1—C8B	−51.8 (4)	C10—N2—C9B—C8B	−30.9 (10)
O4—S2—N2—C10	165.87 (18)	C9A—N2—C9B—C8B	−55.4 (14)
O3—S2—N2—C10	36.5 (2)	S2—N2—C9B—C8B	176.4 (6)
C13—S2—N2—C10	−79.6 (2)	N1—C8B—C9B—N2	−51.2 (11)
O4—S2—N2—C9A	−60.1 (4)	C9A—N2—C10—C11	87.3 (4)
O3—S2—N2—C9A	170.6 (4)	C9B—N2—C10—C11	77.9 (6)
C13—S2—N2—C9A	54.4 (4)	S2—N2—C10—C11	−131.8 (2)
O4—S2—N2—C9B	−39.4 (4)	N2—C10—C11—C12	−65.3 (3)
O3—S2—N2—C9B	−168.8 (4)	C8A—N1—C12—C11	−73.4 (6)
C13—S2—N2—C9B	75.1 (4)	C8B—N1—C12—C11	−84.8 (3)
C6—C1—C2—C3	0.5 (4)	S1—N1—C12—C11	133.74 (19)
C1—C2—C3—C4	−1.8 (4)	C10—C11—C12—N1	62.6 (3)
C1—C2—C3—C7	177.3 (3)	O4—S2—C13—C14	20.1 (2)
C2—C3—C4—C5	1.3 (4)	O3—S2—C13—C14	149.10 (19)
C7—C3—C4—C5	−177.7 (3)	N2—S2—C13—C14	−95.0 (2)
C3—C4—C5—C6	0.4 (4)	O4—S2—C13—C18	−162.33 (19)
C4—C5—C6—C1	−1.7 (4)	O3—S2—C13—C18	−33.3 (2)
C4—C5—C6—S1	178.10 (19)	N2—S2—C13—C18	82.6 (2)
C2—C1—C6—C5	1.2 (4)	C18—C13—C14—C15	1.9 (4)
C2—C1—C6—S1	−178.58 (19)	S2—C13—C14—C15	179.45 (18)
O1—S1—C6—C5	−143.5 (2)	C13—C14—C15—C16	−0.5 (4)
O2—S1—C6—C5	−14.2 (2)	C14—C15—C16—C17	−1.3 (4)
N1—S1—C6—C5	100.1 (2)	C14—C15—C16—C19	176.9 (2)
O1—S1—C6—C1	36.3 (2)	C15—C16—C17—C18	1.6 (4)
O2—S1—C6—C1	165.6 (2)	C19—C16—C17—C18	−176.5 (3)
N1—S1—C6—C1	−80.1 (2)	C16—C17—C18—C13	−0.3 (4)
C12—N1—C8A—C9A	30.8 (12)	C14—C13—C18—C17	−1.5 (4)
C8B—N1—C8A—C9A	56.6 (10)	S2—C13—C18—C17	−179.09 (19)

### Hydrogen-bond geometry (Å, º)

**Table:**

*D*—H···*A*	*D*—H	H···*A*	*D*···*A*	*D*—H···*A*
C10—H10*B*···O3	0.97	2.40	2.886 (3)	110
C12—H12*A*···O1	0.97	2.39	2.878 (3)	111
C10—H10*B*···O4^i^	0.97	2.52	3.142 (3)	122
C12—H12*A*···O2^i^	0.97	2.50	3.035 (3)	115

Symmetry code: (i) *x*−1, *y*, *z*.
